# Caregiver Perceptions of the Transition From Paediatric Intensive to Acute Care

**DOI:** 10.1111/nicc.70662

**Published:** 2026-08-27

**Authors:** Maia D. Regan, Briana P. Keller, Emily Boulos, Kristina Betters, Jessika C. Boles

**Affiliations:** ^1^ Vanderbilt University Nashville Tennessee USA; ^2^ Child Life Department Nicklaus Children's Hospital Miami Florida USA; ^3^ Pediatric Critical Care Department Monroe Carell Jr Children's Hospital at Vanderbilt Nashville Tennessee USA; ^4^ Child Life Department Monroe Carell Jr Children's Hospital at Vanderbilt Nashville Tennessee USA

**Keywords:** family support, family‐centred care, paediatric, transfer, transition

## Abstract

**Background:**

Transfer from the intensive care unit to an acute care unit is reportedly a stressful time for adult patients and their families. However, little has been done to understand these transition experiences and needs in paediatric patients and their parents/caregivers.

**Aim:**

To describe parent/caregiver perceptions of their child's transition from the paediatric general or cardiac intensive care unit to an acute care unit.

**Study Design:**

Using a descriptive electronic survey‐based design, parents/caregivers reported their perceptions of the quality of their child's transition from a paediatric intensive care unit to an acute care unit in a United States hospital, as well as their perceptions about the care received in each location. Responses were analysed using sign tests, Spearman's rank correlations and other non‐parametric tests.

**Results:**

A total of 87 parents/caregivers participated. Participants reported significantly more positive perceptions of the quality of intensive care their child received compared to acute care experiences across all response domains (*z* = −0.3921, *p* < 0.001); this effect held significance even for those with prior paediatric intensive care admissions (*z* = −2.1971, *p* = 0.028). Those who reported receiving adequate preparation and information regarding transfer across units were more likely to report more positive perceptions of overall care quality (*r*(82) = 0.817, *p* < 0.001).

**Conclusions:**

Transferring from the paediatric intensive care unit is a challenging event for paediatric patients and families. With intentional, effective communication and preparation, healthcare providers can empower caregivers as their child's condition improves.

**Relevance to Clinical Practice:**

Families who feel more prepared for transition to acute care are more satisfied with their overall care experience and report that their needs are better met. Intensive care and acute care providers should collaborate on transfer planning initiatives that ensure families are informed, prepared for and supported during transfer between units.

## Impact Statements

- What is known about this topic
  ○ Adult patients and their families identify transfers from intensive to acute care as stressful.
  ○ Medical and psychosocial considerations have been previously examined for adult patients, but not yet for children and their families.
- What this paper adds
  ○ Families in this study generally had more positive perceptions of the care they received in the intensive care unit compared to the acute care unit.
  ○ Caregivers who felt they had been given information about and prepared for unit transfer reported higher satisfaction levels compared with those who felt unprepared.

## 1 Introduction

Over the last decade, admissions to the paediatric intensive care unit (PICU) have increased by approximately 15%, with most of these patients requiring complex and multidisciplinary care spanning days to weeks or longer [1, 2]. Many recent works have demonstrated the vast array of long‐term adverse developmental and psychosocial outcomes observed in patients and families after discharge from the PICU, many of which complicate ongoing efforts to recover physical, cognitive, academic and social/emotional functioning [3, 4, 5, 6, 7, 8, 9, 10]. In response, the current critical care climate now emphasises the ABCDEF bundle as a comprehensive approach to identifying, designing and implementing systematic interventions that may mitigate the long‐term impacts of a PICU stay [3, 11]. Many of these interventions have been well researched and widely adopted, especially those grounded in A (assess pain), B (breathing and awakening trials), C (choice of pain medications and sedating agents), D (assessment and prevention of delirium) and E (early mobility); however, F (family engagement) remains the least examined component of this framework. Without more attention and work in this area, there may be immediate and long‐term effects on the quality and comprehensiveness of care for children and families, and the extent to which the aims of patient‐ and family‐centred care in paediatrics, especially, can be achieved [12].

### 1.1 Background

Many parents/caregivers identify transitions as a significant stressor in all healthcare environments, let alone the intensive care unit, especially as they experience transitions in health status, functional ability and familial routines that previously represented comfort and normalcy. In the adult healthcare literature, transitioning from the intensive care unit (ICU) to a step‐down or acute care unit has been shown to be both a stressor and a risk factor for adult patients and their family members [13, 14, 15]. During this vulnerable period, patients and families must adjust to new treatment regimens, multiple provider teams and loss of routines or supports in the ICU while continuing to navigate the many emotions stirred by the events or illness that prompted ICU admission [13, 14, 15]. From a medical standpoint, intensive‐to‐acute care transitions are also associated with a higher likelihood of medical errors and adverse events [16], which can further elevate family distress, dictate readmission to the ICU and increase the risk of mortality [13, 15, 17, 18]. Existing scholarship in the adult intensive care realm has noted these transitions are often characterised by poor communication, perhaps amplifying uncertainty and confusion regarding the timing of and readiness for transition itself, while trying to help patients and families learn about and navigate the differences in care practices and policies across the intensive and acute care units [13, 19, 20]. Some studies have shown that patients and families are commonly surprised by the lack of one‐to‐one nursing care on step‐down units, resulting in feelings of fear or insecurity as they must adjust to taking on more of the patient's care responsibilities themselves in preparation for discharge [14, 21, 22].

### 1.2 Aim and Hypotheses

Though transition from intensive to acute care is simultaneously a stressor and risk factor for adult ICU patients and their caregivers, research to date has not yet explored this phenomenon in the paediatric domain. More specifically, little is known about caregiver experiences and perceptions during their child's transition from the PICU to an acute care or step‐down unit. Therefore, the purpose of this retrospective study was to describe the care quality perceptions and experiences of parents/caregivers of paediatric patients who transitioned from intensive to acute care.

Based on evidence from adult healthcare, the team hypothesised that parents/caregivers would rate care quality during transition lower than the quality of care received on either type of unit; additionally, it was anticipated that those who reported receiving adequate information and preparation prior to transition would more positively perceive their transfer experience. Regarding differences between groups, it was hypothesised that caregivers of children with a history of prior intensive care stays, or those who were cared for in the paediatric cardiac intensive care unit (PCICU), would report more positive transition experiences and higher overall care quality ratings due to greater familiarity with these processes and continuity of care across the cardiac intensive care and step‐down teams.

## 2 Methods

### 2.1 Design

To describe and explore parent/caregiver experiences and perceptions of intensive‐to‐acute care transition, a retrospective electronic survey‐based design was chosen to ensure a broad geographic reach comparable to the clinical catchment zone of the research site. All data were collected between May 2021 and February 2024.

### 2.2 Setting

Participants were recruited through a non‐profit children's medical centre in the southeastern United States that is affiliated with an academic medical centre. The facility has a 42‐bed tertiary medical paediatric intensive care unit (PICU), a 30‐bed paediatric cardiac intensive care unit (PCICU) and is a certified level I trauma centre serving children with and without private insurance from a three‐state area. Those children who received care in the PICU, by hospital policy, transferred to one of three general paediatric acute care floors once medically stable. Paediatric cardiac intensive care unit patients, on the other hand, transferred to a cardiac‐specific acute care unit on a different side of the same hospital floor. Both intensive care units boasted active and coordinated PICU liberation programs, including systematic implementation of the ABCDEF bundle with emphasis on delirium management and early mobility.

### 2.3 Sample

Those eligible for the study included primary caregivers of children admitted to the PICU or paediatric cardiac ICU at the research site between February 2019 and February 2024. Additional inclusion criteria required participants to be over the age of 18 at the time of participation and speak English proficiently due to limited translation resources for this preliminary study.

Potential participants who met eligibility criteria were sent an informational email or letter from the study team (depending on their stated contact preference in the child's electronic medical record) no less than 15 days after hospital discharge. All participants were clinically cared for during their admission by a member of the study team and were enrolled in the early mobility protocol actively in use across the PICU and PCICU at that time. Additionally, flyers were posted in the acute care units at the research site and outpatient cardiology and pulmonology clinics providing paediatric follow up care after an ICU admission.

### 2.4 Data Collection Tools and Methods

Upon reviewing the recruitment flyer, letter or email, potential participants used the listed hyperlink or QR code to access and complete the study consent form. Next, the participant completed a brief demographic questionnaire about themselves, their child and some details about their child's admission and hospital stay. To maintain patient privacy to the maximum extent possible and to remain in line with expedited‐level Institutional Review Board (IRB) approval at the research institution, these data were collected via self‐report rather than through electronic chart reviews.

The main instrument for this study was the ‘Intensive and Acute Care Experiences and Satisfaction Questionnaire’ designed by the study team. As the aim of this study was to better understand and compare participants' perceptions of the paediatric intensive and acute care environments, and their experiences of transition between the two, extensive literature searches were undertaken to identify any pre‐existing, validated tools for this purpose. However, it was found that such instruments had not yet been created or disseminated; instead, satisfaction, perceived care quality and experience tools that existed were largely connected to adult patient experiences and revolved around environmentally specific aspects of care that could not be applied across both intensive care and acute care environments [23, 24, 25, 26].

In reviewing the literature on parent/caregiver needs during a child's intensive care stay or acute care hospitalisation, four domains of common concerns emerged: perceived quality of care, communication across teams and families, availability of unit resources and honouring of spiritual and cultural needs [27, 28, 29, 30]. Noting these needs, the anchor author employed her 15 years of experience as a Certified Child Life Specialist and psychosocial researcher to design a shortened instrument that could assess participant experiences of these domains in a time‐efficient, accessible and unintrusive way for parent/caregiver participants. This questionnaire was thus composed of three sections: ICU experiences and satisfaction, acute care unit experiences and satisfaction, and transition experiences and satisfaction. Response options included multiple choice, Likert‐type and open‐ended response items (see Appendix S1). The questionnaire was reviewed by and incorporated feedback from a paediatric intensivist, a paediatric hospitalist and the Family Advisory Council at the research site before reaching the final form deployed for this study.

### 2.5 Data Analysis

Each Likert‐style question offered a range of scores from ‘0 = strongly disagree’ to ‘4 = strongly agree’, thus, higher scores indicated higher levels of positive perception or satisfaction. With a total of seven questions pertaining to their intensive care experience, possible scores for this questionnaire section ranged from 0 to 28. As the goal of this study was to identify potential differences rather than scale satisfaction rates, the research team elected not to characterise scores above or below an identified threshold as ‘satisfied’, ‘dissatisfied’ or the like. This type of arbitrary division was thought to be unnecessary for the identified research questions guiding this study.

As the purpose of this study was to describe the care quality perceptions and experiences of parents/caregivers of paediatric patients who transitioned from intensive to acute care, descriptive statistics techniques were planned. Descriptive analyses were conducted using Statistical Package for the Social Sciences (SPSS) version 29. Non‐parametric analyses (sign tests and Mann–Whitney U tests) were used to compare parent perceptions and ratings across intensive to acute settings and between different subgroups, as the final data collected failed to meet normality assumptions as per Shapiro–Wilk tests. Cohen's *d* was used to examine effect sizes. Spearman's rho was also examined to characterise any potential correlations. Results were considered statistically significant at *p* = 0.050.

### 2.6 Ethical and Research Approvals

Expedited‐level IRB approval was obtained from Vanderbilt under protocol number 210700. Ethical and research approval were granted 15 April 2021. All participants provided electronic consent via REDCap before proceeding to the electronic questionnaires housed in the same platform. All data were securely stored in REDCap and an IRB approved, password protected electronic database that only the research team could access. Participant responses were de‐identified, and specific identifiers like the patient's name or medical record number were not collected or requested at any time—nor were electronic charts accessed for any purpose.

## 3 Results

A total of 87 parents/caregivers completed the study (see Table 1 below). Participants were primarily White, non‐Hispanic mothers with greater variability in age, household income and education level. The median length of stay reported in the ICU was 5–10 days, whereas the median length of stay reported for the acute care or step‐down unit was 3–5 days. Sixty‐two percent of participants had a child hospitalised in the general PICU, with the remaining 38% admitted to the PCICU for specialised cardiac intensive care.

**TABLE 1:** Participant demographics.

	Frequency	Percentage (%)
Age		
18–24 years	4	4.6
25–34 years	37	42.5
35–44 years	29	33.3
45–54 years	11	12.6
55–64 years	4	4.6
65 years or older	2	2.3
Gender		
Female	84	96.6
Male	3	3.4
Race		
White	80	93.0
Black or African American	4	4.7
Other	2	2.3
Ethnicity		
Hispanic or Latino	5	5.7
Not Hispanic or Latino	82	94.3
Estimated household income		
Less than $20 000	2	2.3
$20 000–$39 999	13	15.1
$40000–$59 999	12	14.0
$60 000–$79 999	12	14.0
80 000–$99 999	10	11.6
Greater than $100 000	34	39.5
Prefer not to answer	3	3.5
Education		
Less than high school	0	0.0
High school diploma	15	17.2
Technical or trade program	6	6.9
Associate's degree	8	9.2
Bachelor's degree	31	35.6
Master's degree	23	26.4
Professional degree (MD, DO, DDS)	2	2.3
Doctoral degree (PhD)	2	2.3
Religious/Spiritual preference		
Christian	78	89.7
Spiritual	3	3.4
Non‐religious	6	6.9
Child's age		
0–12 months	38	43.7
1–2 years	9	10.3
3–5 years	16	18.4
6–12 years	7	8.0
13+ years	17	19.5
Unit		
PCICU	33	37.9
PICU	54	62.1

*Note:* *N* = 87.

Abbreviations: PCICU, paediatric cardiac intensive care unit; PICU, paediatric intensive care unit.

To describe the care quality perceptions and transition experiences of parents/caregivers of paediatric patients who transitioned from intensive to acute care, comparisons were made across unit ratings. Observed scores for the intensive care perceptions section of the care quality questionnaire were found to range from 0 to 24, whereas observed scores for the same items on the acute care perceptions section ranged from 0 to 28 (see Table 2 below). Further, all individual items on the intensive care portion yielded a higher mean and lower standard deviation than the same items on the acute care section, thereby indicating higher levels of positive perception or satisfaction with the family's intensive care experiences.

**TABLE 2:** Care quality ratings by item and type of unit.

	PICU		Acute care	
	M	SD	M	SD
My family and I had access to the hospital resources we needed to make my child's stay as comfortable as possible.	3.62	0.81	3.38	1.06
There was open, effective and timely communication between my family and the healthcare staff.	3.62	0.81	3.08	1.23
My child's providers spent an appropriate amount of time with my family and I to help us understand our child's illness/injuries and treatment plan.	3.72	0.66	3.20	1.10
My family's cultural needs were met in a respectful and inclusive way during our stay.	3.59	0.76	3.38	0.94
My family's spiritual needs were met in a respectful and inclusive way during our stay.	3.57	0.76	3.37	0.95
My family felt supported by staff during our stay.	3.73	0.66	3.21	1.13
My child received high quality care from the care team.	3.80	0.57	3.20	1.23
Total care quality rating (composite score)	25.66	3.78	22.71	6.45

Abbreviations: M, mean; PICU, paediatric intensive care unit; SD, standard deviation.

To examine differences between composite scores for the intensive care portion of the care quality questionnaire and the acute care section, a sign test was performed. Results indicated a significant difference between composite care quality scores with respect to these two settings (*z* = −0.3921, *p* < 0.001, *d* = 0.44); thus, participants reported more positive perceptions of care quality in the intensive care unit as compared to the acute care unit.

Participants next reported their care quality perceptions with respect to their child's transition from the intensive care unit to the acute care unit (see Table 3 below). Notably, participants gave a mean score of 2.76 (SD = 1.42), out of a possible 5, on the statement ‘my child received the same quality of care in the ICU and step‐down units’. The item regarding receiving ‘adequate information and preparation’ for the child's transfer across units received a mean score of 3.27 (SD = 1.11).

**TABLE 3:** Transition experience ratings.

	M	SD
My family and I were given adequate information about and preparation for our child's transfer from the ICU to the step‐down unit.	3.27	1.11
My child received the same quality of care in the ICU and step‐down units.	2.76	1.42
Please rate your overall satisfaction with transitioning your child's care from the ICU to the step‐down unit.	3.02	1.28

Abbreviations: ICU, intensive care unit; M, mean; SD, standard deviation.

A moderate positive correlation was found between acute care composite care quality scores and having received ‘adequate information about and preparation for our child's transfer’ (*r*(80) = 0.611, *p* < 0.001). All other relationships between composite care quality scores for the intensive care unit, the acute care unit, the transition experience and individual response items were non‐significant.

A moderate positive correlation was found between overall care quality ratings (inclusive of both intensive and acute care) and composite scores on the acute care portion of the questionnaire was noted (*r*(79) = 0.648, *p* < 0.001). This was non‐significant for ICU composite care quality scores (*p* = 0.317). However, there was a strong positive correlation seen between the specific questionnaire item ‘adequate information about and preparation for our child's transfer’ and overall care quality ratings (*r*(82) = 0.817, *p* < 0.001).

There was a weak but significant correlation between responses on the ‘adequate information about and preparation for our child's transfer’ item and ICU composite care quality scores (*r*(82) = 0.227, *p* = 0.038). On the other hand, there was a strong positive correlation observed between composite care quality scores for the acute care unit and a perception that the quality of care on both units was the same (*r*(80) = 0.734, *p* < 0.001). Comparably strong was the positive correlation observed between scores on the ‘adequate information and preparation’ item and scores on the item ‘my child received the same quality of care in the ICU and step‐down units’ (*r*(83) = 0.703, *p* < 0.001).

Interestingly, the sample was relatively well split between participants who had experienced a prior ICU stay with their child (*n* = 46) and those with no prior ICU admissions (*n* = 40). Those participants who reported prior ICU admissions gave significantly higher composite care quality scores on the ICU portion of the questionnaire (*U* = 1513.500, *z* = 2.1651, *p* = 0.030, *d* = 0.233). Similarly, participants who had not experienced a previous ICU stay with their child were significantly more likely to agree or strongly agree that the quality of care on both units was similar (*U* = 1135.500, *z* = 2.196, *p* = 0.028, *d* = 0.24).

Finally, no significant differences were found regarding the type of ICU (PICU or paediatric cardiac ICU) in which the child was hospitalised and composite scores for ICU care quality (*p* = 0.73), composite score for acute care quality (*p* = 0.11), perceptions of receiving adequate transition information (*p* = 0.59), same perceived quality of care between intensive and acute units (*p* = 0.13) or overall care quality ratings (*p* = 0.28).

A summary of key results is shown in Table 4.

**TABLE 4:** Key results summary.

	Composite score (step down)	Adequate transition preparation	Same quality of care across units	History of prior ICU stays
Composite score (intensive care)	*z* = −3.921**	*r*(82) = 0.227*		*U* = 1513.500, *z* = 2.1651*
	*d* = 0.44			
				*d* = 0.233
Overall perceived care quality	*r*(79) = 0.648**	*r*(82) = 0.817**	*r*(83) = 0.703**	
Adequate transition preparation	*r*(80) = 0.611**			
First ICU stay			*U* = 1135.500, *z* = 2.196*	*Mutually exclusive demographic variables*
			*d* = 0.24	
Same quality of care across units	*r*(80) = 0.734**			

Abbreviation: ICU, intensive care unit.

* *p* < 0.05.

** *p* < 0.001.

## 4 Discussion

A paediatric patient and family's transition from the intensive care unit to an acute care or step‐down unit is an event of known medical and psychosocial vulnerability and stress, which has been noted in studies across the United States and multiple European countries [13, 14, 15, 16]. Though prior literature has relegated this phenomenon to adult intensive care unit survivors and their families, the purpose of this study was to describe the perceptions and experiences of parents of children who transferred from the PICU to an acute care or step‐down unit. When asked to reflect on the care they received in these two settings, participants in this study held more positive perceptions of care received in the ICU compared to the acute care unit. Participants indicated more positive endorsements of timely nursing and medical care, cultural and spiritual support for the whole family and effective communication with staff in the ICU as compared to the acute care unit. Moreover, even those families who had experienced one or more previous stays in the PICU with their child perceived higher quality of care in the ICU than the acute care unit. Participants whose child had no history of prior ICU admission, however, were more likely to endorse similar perceptions of care across the ICU and acute care units.

Previous scholarship—including a review of the literature [2]—has suggested that patients and families often build close working relationships with providers on the ICU [1, 13], particularly due to the high level of care in these units and the need for collaborative and constant decision making. The results of this study may demonstrate this same phenomenon, where families who have built more intensive and extended rapport with staff in the ICU may therefore hold more positive perceptions of care quality in that environment and rate their experiences in the acute care unit differently. For those with prior ICU admission histories, this effect could be more pronounced, especially if there is consistency in team members across those ICU admissions and the family therefore has both more rapport and more familiarity in that setting. Regardless of the child's medical history, however, it is crucial that medical and psychosocial staff collaborate to build consistent, communicative and trusting rapport with the patient and family both for the purposes of the current admission and future transfers or readmissions.

Prior literature from the United States [14], Iran [13], Canada [15], Spain [21] and Australia [22] demonstrates that transitions to acute care units are often accompanied by poor communication regarding differences between the units, when a transfer will take place and pertinent details of new treatment regimens. These scholars thus suggest that receiving adequate information about these factors appears to improve the care quality that patients and families perceive. Participants in this study reinforced this presumed relationship, as those who indicated they received adequate preparation and information regarding their child's unit transfer tended to more positively perceive quality of care on the acute care unit. These families also indicated higher levels of agreement with the statement, ‘My child received the same quality of care in the ICU and step‐down units’, and rated higher levels of overall satisfaction compared to families who felt they had not received adequate information about the transition.

Transfer to a new unit, especially after the relatively recent distress associated with a potentially unplanned ICU admission, requires families to anticipate and adapt to change in a time when uncertainty may still be high, and coping resources may be limited by the nature of being in the hospital and away from their community of support. Intensive and acute care units may not only differ in behind‐the‐scenes policies and procedures but also patient‐and‐family‐facing practices; for instance, less face time may be spent with bedside staff on the acute care unit, medication administration may change or take longer (in preparation for upcoming discharge), and families are expected to resume more daily caregiving tasks (like feeding, changing diapers and normal hygiene routines). When families are unprepared for these differences, these changes may cause families to feel even less of a sense of control and competence, which can in turn fuel feelings of fear or perceived lack of safety. This finding is consistent across cultures including the United States [14], Spain [21] and Australia [22].

For participants in this study, transitioning to acute care meant moving to a different unit on the same floor if their child had been in the paediatric cardiac ICU, and moving to a different floor if their child had been in the general PICU. Despite this, no significant differences were found regarding perceptions of care or transition. These findings did not support one of the study hypotheses, and they suggest that experiences of the transition to acute care are not significantly affected by proximity to the ICU where the patient previously stayed.

### 4.1 Limitations

First, this study was conducted using a questionnaire designed by the research team that has not yet been validated as a standardised data collection instrument; it is possible that data collected may not reflect the same level of internal and external validity that would be expected from a validated measure. Despite equitable recruitment methods, most participants in this study were White women who identified as Christian. Thus, the experiences of families who occupy other positionalities may differ, which necessitates additional research. Also, more than 40% of participants reported their child was less than 1 year old at the time of their PICU admission, although this is representative of the children admitted to the research site. Particularly in situations where a firstborn child was admitted to the ICU shortly after birth, caregivers' perceptions of the experience may have been dually affected by trying to adjust to the ICU experience while also adjusting to the experience of being a parent. Future studies could examine differences in care perceptions and experiences in first‐time ICU admissions among those families with and without additional children in the home. Additionally, future work could be done to consider validating the questionnaire used for this study, or to continue developing valid and reliable tools that can shed more insight into parent/caregiver experiences of care quality across these settings and during unit transfers.

### 4.2 Implications for Practice

The results of this study suggest that families who feel more prepared for transition from intensive to acute care are more satisfied with their overall care experience and feel that their needs are more adequately met. To achieve this goal, it is important for ICU and acute care providers to collaborate on transfer planning initiatives that will ensure families are informed, prepared for and supported during transfer between units. As these transitions can occur quickly and with little forewarning, it may be helpful to design a transition planning checklist with staff to ensure patients and families receive pertinent education about unit differences and activities before arriving at the new unit. Healthcare professionals trained in preparing children and families for healthcare encounters such as Certified Child Life Specialists may be an excellent resource for designing and providing this crucial education [31, 32].

## 5 Conclusion

Transitioning from the PICU to the acute care unit can be challenging for patients and families, particularly when preparation and information regarding the transition are seen as inadequate. Though participants in this study perceived care in the ICU more positively as compared to the acute care unit, their overall care perceptions and satisfaction appear to increase when they report receiving adequate information about the transfer across units and are given accurate expectations. Noting the profound psychosocial impact a child's ICU admission can have on children and families, efforts should be made to ensure effective and ongoing communication during all aspects of ICU care—including the transfer to an acute care or step‐down unit—thereby empowering caregivers and preparing them for the next step in their child's medical journey.

## Funding

The authors have nothing to report.

## Disclosure

Generative AI was not used in the creation of this manuscript. All authors have reviewed the final manuscript and are responsible for the manuscript.

## Ethics Statement

This study was approved by the Vanderbilt IRB, protocol #210700, on 4/15/21.

## Consent

The authors have nothing to report.

## Conflicts of Interest

The authors declare no conflicts of interest.

## Supporting information


**Appendix S1:** Questionnaire.

## Data Availability

The data that support the findings of this study are available from the corresponding author upon reasonable request.
