# Supplementary material for: Caregiver Perceptions of the Transition From Paediatric Intensive to Acute Care
Source: Nurs Crit Care. 2026 Aug 27;31(5):e70662. doi: 10.1111/nicc.70662 (PMC13522727; doi:10.1111/nicc.70662)
Supplement: Supplementary file 1 [file NICC-31-0-s001.docx]

**Appendix S1: Questionnaire**

***Section 1: ICU experiences and satisfaction.***

What was the date of your child’s most recent admission to the intensive care unit?

/	/

Which intensive care unit was your child admitted to?

| Pediatric Cardiac Intensive Care Unit (or PCICU, which is typically for children with heart health needs) | Pediatric Intensive Care Unit (or PICU, which is for children with a variety of injuries and illnesses) |
| --- | --- |

How old was your child at that time?

| Less than 12 months | 1 to 2 years | 3 to 5 years | 6 to 12 years | Over 13 years |
| --- | --- | --- | --- | --- |

How many days did your child spend in the intensive care unit?

| Less than 24 hours | 1 to 2 days | 3 to 5 days | 5 to 10 days | More than 10 days |
| --- | --- | --- | --- | --- |

What was the reason for your child’s admission to the intensive care unit?

How many intensive care unit admissions has your child had, including this most recent experience?

| 1 | 2 | 3 | 4 | 5 | 6 to 10 | More than 10 |
| --- | --- | --- | --- | --- | --- | --- |

Please answer the following questions specifically about you and your child’s stay in the intensive care unit.

|  | Strongly Agree | Somewhat Agree | Neutral | Somewhat Disagree | Strongly Disagree |
| --- | --- | --- | --- | --- | --- |
| My family and I had access to the hospital resources we needed to make my child's stay in the intensive care unit as comfortable as possible. | ☐ | ☐ | ☐ | ☐ | ☐ |
| There was open, effective, and timely communication between my family and the healthcare staff during our intensive care stay. | ☐ | ☐ | ☐ | ☐ | ☐ |
| My child's intensive care providers spent an appropriate amount of time with my family and I to help us understand our child's illness or injuries and treatment plan. | ☐ | ☐ | ☐ | ☐ | ☐ |
| My family's cultural needs were met in a respectful way during our intensive care stay. | ☐ | ☐ | ☐ | ☐ | ☐ |
| My family's spiritual needs were met in a respectful and inclusive way during our intensive care stay. | ☐ | ☐ | ☐ | ☐ | ☐ |
| My family felt supported by staff during my child's intensive care stay. | ☐ | ☐ | ☐ | ☐ | ☐ |
| My child received high quality care from the intensive care team. | ☐ | ☐ | ☐ | ☐ | ☐ |

***Section 2: Acute care unit experiences and satisfaction.***

How many days did your child spend on the acute care unit, after transferring from the ICU?

| Less than 24 hours | 1 to 2 days | 3 to 5 days | 5 to 10 days | More than 10 days |
| --- | --- | --- | --- | --- |

What type of acute care unit was your child transferred to after their most recent intensive care stay?

| General Medicine | Hematology/Oncology | Cardiology | Surgery/Trauma | Other | I don’t know |
| --- | --- | --- | --- | --- | --- |

Please answer the following questions specifically about you and your child's stay on the step-down (or acute care) unit. For the purposes of this questionnaire, the term "step-down unit" refers to any unit other than intensive care in which your child was hospitalized after their time in the ICU.

|  | Strongly Agree | Somewhat Agree | Neutral | Somewhat Disagree | Strongly Disagree |
| --- | --- | --- | --- | --- | --- |
| My family and I had access to the hospital resources we needed to make my child's stay in the step-down unit as comfortable as possible. | ☐ | ☐ | ☐ | ☐ | ☐ |
| There was open, effective, and timely communication between my family and the healthcare staff during our stay in the step-down unit. | ☐ | ☐ | ☐ | ☐ | ☐ |
| My child's step-down unit providers spent an appropriate amount of time with my family and I to help us understand our child's illness or injuries and treatment plan. | ☐ | ☐ | ☐ | ☐ | ☐ |
| My family's cultural needs were met in a respectful way during our step-down unit stay. | ☐ | ☐ | ☐ | ☐ | ☐ |
| My family's spiritual needs were met in a respectful and inclusive way during our step-down unit stay. | ☐ | ☐ | ☐ | ☐ | ☐ |
| My family felt supported by staff during my child's step-down unit stay. | ☐ | ☐ | ☐ | ☐ | ☐ |
| My child received high quality care from the step-down care team. | ☐ | ☐ | ☐ | ☐ | ☐ |

***Section 3: Transition experiences and satisfaction.***

Please answer the following questions regarding you and your child’s overall hospital stay.

|  | Strongly Agree | Somewhat Agree | Neutral | Somewhat Disagree | Strongly Disagree |
| --- | --- | --- | --- | --- | --- |
| My family and I were given adequate information about and preparation for our child’s transfer from the ICU to the step-down unit. | ☐ | ☐ | ☐ | ☐ | ☐ |
| My child received the same quality of care in the ICU and step-down units. | ☐ | ☐ | ☐ | ☐ | ☐ |

Please rate your overall level of satisfaction with transitioning your child’s care from the ICU to the step-down unit.

| Very Satisfied | Somewhat Satisfied | Neither Satisfied nor Dissatisfied | Somewhat Dissatisfied | Very Dissatisfied |
| --- | --- | --- | --- | --- |

Please tell us about any ways in which you feel we could better support families when transitioning from the intensive care unit to a step-down unit.
